# Relationships between Plasma Concentrations of Testosterone and Dihydrotestosterone and Geriatric Depression Scale Scores in Men and Women Aged 60–65 Years—A Multivariate Approach with the Use of Quade’s Test

**DOI:** 10.3390/ijerph191912507

**Published:** 2022-09-30

**Authors:** Kamil Karolczak, Joanna Kostanek, Bartlomiej Soltysik, Lucyna Konieczna, Tomasz Baczek, Tomasz Kostka, Cezary Watala

**Affiliations:** 1 Department of Haemostatic Disorders, Medical University of Lodz, ul. Mazowiecka 6/8, 92-215 Lodz, Poland; 2 Department of Geriatrics, Healthy Aging Research Center (HARC), Medical University of Lodz, pl. Hallera 1, 90-647 Lodz, Poland; 3 Department of Pharmaceutical Chemistry, Medical University of Gdańsk, ul. Hallera 107, 80-416 Gdańsk, Poland

**Keywords:** testosterone, dihydrotestosterone, sex, depression, risk factor, geriatric scale of depression

## Abstract

The potential role of testosterone and dihydrotestosterone in the pathogenesis of depression in older subjects is poorly recognized and understood. The current study examines the symptoms of depression in males and females at the age of 60–65 using a short version (15 questions) of the Geriatric Depression Scale (GDS) questionnaire. Blood plasma levels of androgens were estimated by LC/MS/MS. Total GDS score calculated for males were not found to be significantly associated with plasma levels of testosterone or dihydrotestosterone. Older men with higher plasma testosteronemia were more likely to report being in good spirits most of the time, but more willing to stay at home than undertake outside activities. The men with higher plasma levels of dihydrotestosterone also perceived themselves as being in good spirits most of the time. Older men with higher testosterone were more likely to report having more problems with their memory than others. No significant associations were found between plasma levels of androgens and GDS scores in older women; however, some tendencies suggest that testosterone and dihydrotestosterone may act as antidepressants in older women.

## 1. Introduction

Old age is a significant risk factor for the development of depression. Unfortunately, the popular acceptance of depression as a “normal” part of aging or even a “natural” feature of elderliness is a serious obstacle to diagnosing and treating aging-associated depression. The widely held assumption that a depressive mood may be a part of aging is believed to lower reporting of mood disorders by older subjects. Plus, families, caregivers and physicians often neglect or underestimate the importance of mood disorders in older family members and patients. Importantly, aging-associated depression is not exactly the same phenomenon as depression in younger people [1], and despite the partial overlapping of certain symptoms, significant differences can be noted regarding the course and prognosis of depression in older subjects [2,3,4]. Thus, age-related depression is a challenging medical problem.

The main risk factors for the development of depression include aging [5], sex (women seem to be more prone [6]), negative subjective health assessments and poor health status [7,8], coexistence of chronic diseases [7], disability [9] or sleep disturbances [10]. In addition, the risk can be influenced by the marital status, although this is controversial [11,12], and by the area of residence, with higher risks noted both in urban [11] or rural areas [13] depending on the study. The education status and family income were negatively associated with the severity of depression [11,13]. Plus, a lack of hobbies, abandoning regular involvement in daily activities and abandoning regular religious practice are social markers of increased risk of depression [13], as well as a lack of employment and lack of health insurance, together with being dependent [12]. However, the physical health status and the presence of comorbidities serve as the greatest risk factors [14].

As well as presenting a serious medical problem in terms of diagnosis and treatment, depression in the elderly also represents a serious biological challenge. Few very plausible biological factors have been identified as significantly correlated with the risk of developing depression among the elderly. Among these, inflammation, and particularly pro-inflammatory cytokines, have been noted [15], together with immune activation [16] and impaired neurogenesis [17].

To the biological factors shaping the risk of depression in older subjects we can also add, on the basis of a current scientific literature, the hormonal status, with a special regard to sex steroids, and particularly androgens. In men, testosterone levels gradually decline with age [18]; up to 30% of men aged 60 years and older have a total testosterone concentration below 350 ng/dL, and free testosterone below 225 pmol/L [19]. Testosterone has already been suggested as an anti-inflammatory hormone [20] able to enhance the survival of newly-generated neurons [21]. Both neuroinflammation [22] and alterations in neurogenesis [23,24] have been proposed as probable key factors contributing to depression. Lower concentrations of free testosterone have been associated with a predisposition to depression [25]; however, the evidence remains ambiguous [26,27].

It has recently been proposed that testosterone may play a potentially significant role in the health of postmenopausal women [28]. Women with severe major depression and concomitant hypercortisolemia exhibited higher blood levels of testosterone and dihydrotestosterone, and the women from this group did not demonstrate an age-associated decline in testosterone levels, unlike healthy controls [29]; this clearly suggests that testosterone may increase the risk of depression in older women. Free testosterone may be a factor increasing the risk of depression in overweight, pre-obese and obese postmenopausal women when compared to non-depressive controls (significantly higher levels of free testosterone were found in postmenopausal pre-obese, obese and overweight individuals) since hyperandrogenism associates positively with the accumulation of abdominal fat, which in turn, seems to associate directly with an increased risk of depression in women [30]. Thus, the potential role of androgens in determining the risk of depression is an even more challenging subject in women than in men.

The most urgent issue is to find the most efficient tool (or tools) for the early detection of depression or for the forecasting of the development of mood disorders in older subjects. Such a tool must be capable of recognizing the multifactorial genesis of depression. One intriguing solution is based on a combination of measurements of testosterone and dihydrotestosterone in blood plasma, i.e., biological factor(s) associated with the risk of depression, and the geriatric depression scale (GDS) questionnaire for tracking the non-biological signs of depression. The GDS is a universal and commonly used diagnostic tool that has been already successfully applied for the assessment of depressive symptoms among older subjects from various ethnic, socioeconomic and clinical backgrounds [31,32,33,34,35,36].

To better understand the role of testosterone and dihydrotestosterone in the development of depression, the present study examines the associations between androgen (testosterone and dihydrotestosterone) concentrations in the blood plasma and the results of the geriatric depression scale (GDS) in men and women aged 60–65 years.

## 2. Materials and Methods

### 2.1. Chemicals

LC-MS-grade acetonitrile (MeCN), methanol (MeOH), tetrahydrofuran (THF), MS-grade formic acid (HCOOH), analytical standard testosterone and dihydrotestosterone (minimum purity of 99%) and dimethyl sulfoxide were delivered by Sigma (St. Louis, MO, USA). Nitric acid (HNO_3_) was purchased from POCH (Gliwice, Poland). HPLC-grade dichloromethane (DCM) was provided by VWR (Radnor, PA, USA). PBS was sourced from Avantor Performance Materials Poland S.A. (Gliwice, Polska).

Ultrapure water was obtained from a Milli-Q purification system (Millipore, Bedford, MA, USA). Nitrogen (NM32LA Nitrogen Generator, Peak Scientific Instruments, Billerica, MA, USA) was used as a drying gas.

### 2.2. Study Population

All steps of the experiments with the participation of human subjects were undertaken under the guidelines of the Helsinki Declaration for Human Research following approval by the Committee on the Ethics of Research in Human Experimentation, Medical University of Lodz. A written summary of the experiment, including detailed information regarding the study objectives, study design, risks and benefits, was presented to all volunteers during recruitment. Written informed consent was obtained from each individual before the beginning of the experiments. All recruitment stages were performed according to a previously described protocol [37].

This study was performed as a part of the “Occurrence of oxidative stress and selected risk factors for cardiovascular risk and functional efficiency of older people in the context of workload” project supervised by the Clinic of Geriatrics at the Medical University in Lodz (Lodz, Poland) and funded by the Central Institute For Labour Protection-National Research Institute (Warsaw, Poland). The participants responded to announcements given on local TV, radio and newspapers. The inclusion criteria comprised the following: aged from 60 to 65 years and willing to participate. The recruitment process is described more clearly elsewhere [37].

The research group included 300 subjects (150 men and 150 women), aged from 60 to 65 years (group-matched age distribution). Of the enlisted target population, 71 men and 79 women, who had a complete list of laboratory results, were selected by simple unrestricted randomization. In some analyses, propensity score matching (PSM) was used to ensure a balanced age-matched sex distribution (*n* = 142, 71 men and 71 women). Any participants who declared current use of antidepressants were not included in the final groups.

### 2.3. Blood Sampling, Isolation of Blood Plasma, Measurements of Blood Morphology and Serum Biochemistry

Blood was collected by aspiration to vacuum tubes (Sarstedt, Nümbrecht, Germany). This was supplemented with 0.105 mol/L buffered sodium citrate (citrate:blood ratio = 1:9, *v*/*v*) in samples used for androgen measurements or with EDTA for blood morphology samples. No anticoagulant was used for blood samples prepared for serum isolation. Blood was collected from a peripheral vein cannulated with an 18-gauge needle after overnight fasting.

The blood morphology parameters were measured with a 5-Diff Sysmex XS-1000i the hematological analyzer (Sysmex, Kobe, Japan), while serum biochemical parameters were evaluated with a DIRUI CS 400 analyzer (Dirui, Changchun, China).

To obtain blood serum, the whole blood, without anticoagulant, was incubated for 30 min at 37 °C and centrifuged (2000× *g*/15 min/4 °C). The supernatant (serum) was aspirated and used for further analysis.

Blood plasma was obtained from citrated blood, centrifuged immediately after blood sampling (1000× *g*/15 min/4 °C), portioned (1 mL), immediately frozen and kept at −80 °C. The samples were used within six months.

### 2.4. HPLC-MS

HPLC/MS/MS measurements of testosterone and dihydrotestosterone concentrations in blood plasma were performed as described earlier [38].

### 2.5. Geriatric Scale of Depression

A normal mood or mild or deep depression was identified using the short version of the Geriatric Scale of Depression (GDS) questionnaire, comprising 15 yes/no questions. The total score indicates the likelihood of depressive symptoms and the degree of depression. The questionnaire comprises the following: 1. Are you basically satisfied with your life? 2. Have you dropped many activities or have your interests diminished? 3. Do you feel that your life is empty? 4. Do you often get bored? 5. Are you in good spirits most of the time? 6. Are you afraid that something bad is going to happen to you? 7. Do you feel happy most of the time? 8. Do you often feel helpless? 9. Do you prefer to stay at home, rather than going out and doing things? 10. Do you feel that you have more problems with your memory than most? 11. Do you think it is wonderful to be alive now? 12. Do you feel worthless the way you are now? 13. Do you feel full of energy? 14. Do you feel that your situation is hopeless? 15. Do you think that most people are better off than you are?

### 2.6. Statistical Analysis

Data are presented as the mean ± SD or median (Me) and interquartile range (IQR: LQ [25%] to UQ [75%]). The normality of the distribution for continuous variables was tested with the Shapiro–Wilk test, and variance homogeneity with Levene’s test. Two-group comparisons for continuous variables were performed with Student’s *t*-test or the Mann–Whitney *U* test, depending on whether the assumptions of normality and homogeneity were met. Discrete (non-continuous) variables were tested using either the Mann–Whitney *U* test, chi^2^ test or Fisher’s exact test. The GDS responses were compared between men and women with adjustment of confounders (age, sex) using bootstrap-boosted multivariate logistic regression (women constituted the reference class, men the modeled class; V-fold cross-validation). The goodness-of-fit of the multivariate logistic regression models was verified with a Hosmer–Lemeshow test. The associations between the concentrations of Tst and DHT and the dichotomic values of the GDS scores were analyzed with various nonparametric techniques. 

In addition to the gamma rank correlation test and biserial point correlation, the study used a new technique, first reported by Quade, to calculate nonparametric partial correlation coefficients [39,40]. The technique was designed to estimate nonparametric (order-scale, qualitative) correlations by generalizing this class of correlations to partial correlations. Some analyses, including Quade’s procedure, used propensity score matching (PSM) to ensure the balanced age-matched proportions of both sexes, while other procedures used an adjustment for age and sex. The following software were used for statistical analyses: Statistica v. 13.1 (Statsoft, Dell Inc., Tulsa, OK, USA), StatsDirect v. 3.0.182 (StatDirect Ltd., Birkenhead, Wirral, UK), Resampling Stats Add-In for Excel v. 4 (The Institute for Statistics Education, An Elder Research Company, Charlottesville, WA, USA) and R Package Software v. 4.4 (a program for calculating Quade’s coefficients written by one of the authors; J.K.).

## 3. Results

### 3.1. Medical, Anthropometric and Social Characteristics of the Studied Groups of Participants

The mean age of all volunteers tested in the study was 62.5 years (±1.7); this age differed significantly between men (62.9 ± 1.7) and women (62.6 ± 3.5) (*p* < 0.05 in the non-paired Student’s *t*-test). The average BMI (kg/m^2^) calculated for men and women was similar (Me = 27.5 (IQR: [25.1–30.4]) for women and Me = 27.2 (IQR: [24.4–30.04]) for men (*p* > 0.05 in the Mann–Whitney *U* test); the mean BMI for the whole group was 27.3 [24.7–30.3].

The participants reported the following diagnosed diseases: hypertension (47% in both sexes; 54% in men and 39% in women), hypercholesterolemia (63% in both sexes; 59% in men and 66% in women), type 2 diabetes mellitus (9% in both sexes; 11% in men and 7% in women), previous myocardial infarction (1.5% in both sexes; 2.0% in men and 0.8% in women), previous stroke (2.5% in both sexes; 3% in men and 2% in women), osteoporosis (11% in both sexes; 2% in men and 20% in women), diseases of the digestive tract (33% in both sexes; 27% in men and 39% in women), previous cancer (7% in both sexes; 6% in men and 7% in women), ophthalmologic diseases (20% in both sexes; 19% in men and 20% in women), depression (15% in both sexes; 10% in men and 20% in women), chronic obstructive pulmonary disease (13% in both sexes; 11% in men and 14% in women), joint diseases (48% in both sexes; 44% in men and 52% in women).

In addition, 24% of the men and 22% of the women reported smoking at the time of investigation. The following drugs were being taken during the study: nitrates—0.4% in both sexes (0.8% of males (M), 0% of females (F)); beta blockers—19.5 in both sexes (19% M, 20% F), angiotensin-converting enzyme inhibitors—18.2% in both sexes (18% M, 19% F), calcium channel blockers—10.6% in both sexes (14% M, 7% F), indapamide—11% in both sexes (13% M, 9% F), sartans—5.7% in both sexes (5% M, 6.5% F), alpha blockers—5.7% in both sexes (11.3% M, 0% F), statins—17% in both sexes (19% M, 15% F), insulin—2.4% in both sexes (5% M, 0% F), metformin—6.5% in both sexes (6.5% M, 6.6% F), gliclazide/glimepiride—3.7% in both sexes (5.6% M, 1.6% F).

Other main medical markers like blood count and blood biochemistry are shown in Table 1.

### 3.2. Selection of Age-Matched Sex Subgroups with Propensity Score Matching Algorithm (PSM)

Participants were selected for the two sex subgroups by multivariate logistic regression, taking sex as the dependent variable, and age, use of antidepressants and use of steroid drugs as independent variables. The resulting propensity scores were used to select two age-matched sex subgroups based on the propensity score matching (PSM) method using the nearest neighbor with no replacement algorithm. The groups were selected based on the greatest possible similarity of cases between groups, with respect to age and drugs used. Each group contained 73 individuals. The resultant median age and propensity score values, as well as the fractions of drug used, were: 63, 61–64 years vs. 61, 63–65 years in women and men, respectively, *p* = 0.084; 0.497, 0.411–0.552 vs. 0.502, 0.411–0.587 for PS in women and men, respectively, n.s., both by the Mann–Whitney *U* test; 2.74% vs. 2.74% for the use of antidepressants in women and men, respectively, n.s.; and 0 vs. 0 for the use of steroid drugs, n.s., both by Fisher’s exact test.

### 3.3. Geriatric Depression Scale Scores Depend on Sex and Steroid Hormones

Multivariate logistic regression revealed a few statistically significant associations between sex and the total score for questions 1 (Are you basically satisfied with your life?), 3 (Do you feel that your life is empty?), 4 (Do you often get bored?), 6 (Are you afraid that something bad is going to happen to you?) and 8 (Do you often feel helpless?). However, following additional adjustment for TST or DHT concentrations, these sex-based associations only remained significant for questions 1 and 6 (Supplementary Table S1).

We also calculated the differences in plasma TST and DHT levels in men and women responding “yes” or “no” to particular questions on the GDS scale that they scored one point on, and we noted no significant differences for any GDS questions except for question 2 (“Have you dropped many activities or have your interests diminished?” for Tst and DHT in both sexes, and for DHT in females) (Supplementary Table S2).

We observed that a higher percentage of men scored one point on questions 1 (Are you basically satisfied with your life?), 7 (Do you feel happy most of the time?), 9 (Do you prefer to stay at home, rather than going out and doing things?), 11 (Do you think it is wonderful to be alive now?) and 13 (Do you feel full of energy?). For questions 10 (Do you feel that you have more problems with your memory than most?) and 12 (Do you feel worthless the way you are now?), the percentage of men and women scoring one point was identical or almost identical. A greater percentage of women obtained one point with their answers to questions 2 (Have you dropped many activities or have your interests diminished?), 3 (Do you feel that your life is empty?), 4 (Do you often get bored?), 5 (Are you in good spirits most of the time?), 6 (Are you afraid that something bad is going to happen to you?), 8 (Do you often feel helpless?), 14 (Do you feel that your situation is hopeless?) and 15 (Do you think that most people are better off than you are?) (Table 2).

The comparison of the frequencies of the obtaining of one point in response to individual questions of the geriatric depression quaestionnaire revealed that there is a statistically significant relationship between the sex (with higher frequencies of responses suggesting depression in women) and GDS score for questions 1, 2, 3, 4, 6, 7 and 8 (Table 3). The outcomes of the chi-squar/Fisher’s exact test analyses (Table 3) were confirmed by odds ratios following additional adjustments for age and concentrations of steroid hormones for questions 1 and 6 (Supplementary Table S1).

Men were characterized by a lower overall GDS score (Me = 2; IQR: 1–3) than women (Me = 3; IQR: 1–5, *p* < 0.001 in the Mann–Whitney *U* test).

### 3.4. Associations between Plasma Levels of Testosterone and Dihydrotestosterone and Geriatric Depression Scale Scores in Men and Women

In women, plasma testosteronemia we found a statistical tendency for the associations between the GDS score for questions 1 (Are you basically satisfied with your life?), 8 (Do you often feel helpless?) and 10 (Do you feel that you have more problems with your memory than most?), and plasma dihydrotestosteronemia with GDS question 2 (Have you dropped many activities or have your interests diminished?) (Table 4).

In men, a significant positive association was found between plasma testosteronemia and the GDS scores for question 10 (Do you feel that you have more problems with your memory than most?). In addition, the plasma TST and DHT concentrations were negatively correlated with the score for question 5 (Are you in good spirits most of the time?), while the TST concentration, but not DHT, was positively correlated with the score for question 9 (Do you prefer to stay at home, rather than going out and doing things?) (Table 4).

In all participants, the plasma TST and DHT concentrations were significantly negatively associated with the total scores for questions 4 (Do you often get bored?), 8 (Do you often feel helpless?), 14 (Do you feel that your situation is hopeless?) and the total GDS score, but only concentration of DHT was significantly and negatively associated with the total scores for 15 (Do you think that most people are better off than you are?). The plasma DHT concentration demonstrated a statistical tendency for negative association with the scores for questions 2 (Have your interests diminished?) and 3 (Do you have a feeling of emptiness in your life?). For both TST and DHT we noted a statistical tendency for negative association with the total score for question 5 (Are you in good spirits most of the time?) (Table 4).

Point-biserial correlation analysis showed that both TST and DHT levels were significantly negatively associated with the scores for questions 4 (Do you often get bored?), 8 (Do you often feel helpless?) and 14 (Do you feel that your situation is hopeless?). Both tested androgens were positively associated with the scores for question 11 (Do you think it is wonderful to be alive now?). In the case of question 3 (Do you feel that your life is empty?) we noted only statistical tendencies for both androgens.No significant association was found between the TST or DHT level and the scores obtained for the remaining questions (Table 5). In addition, point-biserial correlation analysis indicated that TST and DHT levels were negatively associated with the total scores for respondents of both sexes (data not shown).

### 3.5. Quade’s Index for the Correlation of the Hormone with the Total GDS Score in the Group of Males and Females

Using Quade’s index has a great advantage over the rank gamma correlation or biserial correlation because it can be used to estimate the nonparametric measures of “partial correlation”. The non-adjusted indices and the indices adjusted for sex, as well as the gamma correlation coefficients calculated for the original data (not age-matched original groups of men and women after exclusion of those using antidepressant drugs) and for the age-matched data (PSM algorithm), after adjusting them to the original sample size of *n* = 150, are presented in Table 6. In the total group (not age-matched), the plasma concentrations of TST and DHT were found to be significantly negatively associated with the total GDS score (Table 6, gamma correlation, not age-matched). However, Quade’s indices calculated for the PSM-age-matched group (equal proportions of men and women) were not significant, regardless of whether they were adjusted for sex (Table 6, Quade’s index, not adjusted/adjusted for sex). Interestingly, when using the gamma correlation coefficients calculated for the data matched for age using the PSM algorithm, the correlations for TST and DHT with the total GDS score remained negative but were not statistically significant (Table 6, gamma correlation, age-matched).

Considering that the subgroups of men and women were not very large, the above calculations were performed using bootstrap-boosted procedures (10,000 iterations) to ensure that the final outcomes were not influenced by pure chance. The outcomes confirm that the statistically significant associations noted between TST and DHT plasma levels and total GDS scores were observed merely for non-age-matched data. Hence, it is not the sex differences per se, but rather the subtle, very significant age differences between men and women, which appear to be responsible for the significant associations between TST, DHT and the total GDS score.

## 4. Discussion

Our findings indicate that men with higher plasma levels of testosterone and dihydrotestosterone are more likely to be in good spirits most of the time, suggesting that androgens have an antidepressive action in men. However, male responders with higher plasma testosteronemia were more willing to stay at home instead of going out. Plasma dihydrotestosterone levels did not appear to be related to this mood characteristic in men. It also should be mentioned that older men with higher testosterone levels showed a significant tendency to report experiencing more problems with memory. Thus, testosterone may have some pro and antidepressive action among older males.

No statistically significant associations were found between plasma levels of androgens and any depressive characteristics in women. Only a few significant tendencies were found, which suggest that testosterone has antidepressive potential in older women, since those with higher plasma testosterone were more often satisfied with life, rarely felt helpless and rarely reported more problems with memory than most. Plus, women with higher plasma levels of dihydrotestosterone were less likely to forego activities and interests compared to those with lower levels, suggesting they may be less prone to depression. The results presented by us contradict previous outcomes suggesting a prodepressive action of testosterone in women [29]. Yet, we should be aware that Weber et al. [29] presented results for both pre- and postmenopausal women, whereas we presented data exclusively for postmenopausal females. Our results showing some tendencies for there to be a lower risk of depression in older women with higher levels of androgens are in contradiction with the results of Stanikova et al. [30], who found no changes in total or free testosterone in postmenopausal women with depression in comparison to healthy controls. However, since the associations presented herein are only statistical tendencies, further research is needed with larger groups of female participants.

In the mixed group, i.e., both men and women, the testosterone and dihydrotestosterone levels were significantly negatively associated with boredom, helplessness and hopelessness. Only dihydrotestosterone appeared to be negatively associated with feeling that most people are better off.

Plus, some statistical tendencies indicated that both men and women with higher plasma dihydrotestosteronemia were less likely to forego many activities and interests, and to experience feelings of emptiness. Those with higher levels of both androgens more often reported being in good spirits most of the time. Hence, it appears that both androgens affect different aspects of anti/pro-depressive states in older men.

Moreover, both the testosterone and dihydrotestosterone levels were negatively correlated with the total GDS score, suggesting that they generally protect against depression in older people. However, this conclusion is based on our pooled data, where a single common group, including both men and women, was analyzed with Spearman’s rank correlation test. These findings were not supported by Quade’s index. As analyses performed in separate sex subgroups seem to yield more accurate results, it is possible that both androgens shape some aspects of depression risk, reflected by individual questions in the GDS; these may contribute to the risk of depression in a sex- and androgen-specific manner, but are not related to the total GDS score evaluated in men or women.

Although the GDS is a popular and useful tool, it is not the only one [41,42,43] for screening depressive symptoms among older people; nonetheless, it supports the reliable identification of moderately and severely depressed subjects [44]. It remains unclear whether plasma androgen levels may be used as biological markers of the risk of depression as few studies have examined the relationship between testosterone levels and mental symptoms such as depression and mood disturbances in the elderly [45,46,47]. As a result, they are currently far from being recognized as clinically useful diagnostic predictors of age-associated depression. However, the literature indicates that testosterone exhibits antidepressive features. Cognitive performance, manifested as inter alia, memory, verbal fluency, visuospatial orientation and attention, is significantly poorer in men with lower concentrations of testosterone and can be improved by testosterone supplementation [45]. Depression among men appears to be significantly associated with blood testosteronemia [46,48,49,50]. Moreover, treatment with testosterone, especially higher doses, is well-tolerated and brings significant clinical improvement in subjects with less variable symptoms [45]. Men with prostate cancer demonstrate a significantly higher risk of depressive symptoms following pharmacological androgen deprivation [51,52], further indicating that testosterone shapes a positive mood.

These findings are in line with those of the Amirkola Health and Aging Project (AHAP), which indicate that the number of episodes of depression is negatively correlated with the blood testosterone concentration. This is of great interest as the project used a similar methodology to the present study. The AHAP project, a relatively large study involving 830 men [53], used a short version of the GDS scale (15 questions) to diagnose depression, and used commercially available ELISA kits to measure the levels of testosterone. Their findings indicate significantly lower levels of testosterone in men exhibiting depression. The authors note that lower testosterone remained a significant factor increasing the risk of depression, even after adjustment for the educational status and living alone.

Contrary to the AHAP study [53], the present study measured the testosterone level using LC-MS: the “gold standard” for androgen analysis in biological samples. LC-MS offers high sensitivity and specificity (especially at low analyte concentrations), as well as high throughput and limited requirements for sample preparation. While ELISA tests are easy to perform, they are at risk of cross-reactions, resulting in false-positive results. Caution should be exercised when using ELISA, especially in direct tests where there is no matrix purification step; in addition, only one analyte can be measured. In contrast, the LC-MS method obtains more reliable results.

A study of a wide age range (18–65) of women found patients with currently diagnosed depression to have significantly lower levels of salivary testosterone than control groups. Plus, significantly lower salivary testosterone levels were noted in women with social phobia, agoraphobia and generalized anxiety disorders [54]. Stanikova and co-workers found depressive symptoms to be associated with a higher BMI, but only in the premenopausal subgroup, in which the pro-depressive effects of BMI were clearly mediated by testosterone [30]. Unfortunately, little research has been performed on the influence of testosterone on depression in women, and further studies are needed in this area to form definite conclusions.

Interestingly, a deeper molecular insight into the antidepressive action of androgens has been provided by studies on women. It has been discovered that testosterone increases the concentration of GABA in the posterior cingulate in young (av. 30) women with depressive syndrome [55]; however, the study was performed on young women of reproductive age, and as the nature of depression varies with age, the results should be extrapolated with caution [3]. Unfortunately, the possible involvement of GABA in the pathogenesis of depression remains unclear, and hence the possible mediation of the antidepressive action of testosterone through GABA remains to be tested [55].

Studies suggest that the reduced forms of testosterone, such as 3α-androstenediol or androsterone, are not mediators of testosterone-induced anxiolysis [56].

The amygdala, hypothalamus, hippocampus and neocortex appear particularly sensitive to the action of testosterone [57,58,59,60]. Therefore, these regions may be preferred sites of androgen binding, including in the present subjects. It may be that changes in the androgen receptor expression may influence the pathogenesis of depression. Indeed, subjects with depression have demonstrated lower androgen receptor mRNA expression in the paraventricular nucleus (men and women, mean age of 67 years) [61]. Hence, it is possible that our participants showing significant associations between plasma (dihydro) testosteronemia and some aspects of depressive behavior and mood may have been subject to considerable androgen signaling dysregulation in the brain.

The present study has some limitations. A considerable number of biological factors are known to influence the risk, intensity and course of depression [3]. Among these, the plasma concentrations of androgens studied herein represent only a single, relatively newly recognized example, and our present study makes no reference to other potential markers in the blood [62] or neuroimaging [63,64]. Some previous studies also suggested that the body composition may be pivotal in mediating the effects of testosterone in depression, which implies that testosterone is not independently associated with depressive symptoms [65] and should be considered in a net of other contributing factors.

The results presented herein blend brilliantly with the wider trend of the worldwide discussion on the role of androgens in shaping the health of older men and women. The increasing number of results indicate the beneficial effects of testosterone in shaping the general wellbeing, mood, lipid profile, glucose metabolism and fat mass reduction, and increases in muscle mass and tissue sensitivity to insulin. All these benefits stimulated by testosterone raise high hopes, and it is believed that testosterone replacement therapy in hypogonadal men (usually characterized by a depressed mood, unfavorable glycolipid profile, insulin resistance and obesity) can reduce the risk of many undesirable consequences. In fact, many clinical studies and meta-analyses have confirmed such possibilities. Unfortunately, it is still controversial as to what extent testosterone replacement therapy can reduce or increase the risk of cardiovascular events. There is also an extremely interesting discussion on the effect of testosterone replacement therapy on the risk of developing prostate cancer. It remains to be seen whether in the coming years these controversies will be solved by the findings of planned and fully controlled research studies [66].

Today, it cannot be confidently claimed that plasma levels of androgens present clear advantages over other approaches in screening for the risk of depression. Further studies including multifactorial analyses are needed to obtain a full and clear picture of the risk of depression.

## 5. Conclusions

Androgens were found to correlate only with some of the symptoms of depression tracked by the GDS questionnaire; as such, it is doubtful that our approach is of value. Nevertheless, as previous studies have found androgens to be reasonable candidates as markers of depression, this issue merits further research in larger studies.

## Figures and Tables

**Table 1:** Blood morphology and plasma/serum biochemistry noted in the studied group.

Variable	Both Sexes (*n* = 150)	Males (*n* = 71)	Females (*n* = 79)
*Indices of blood morphology and biochemistry*			
WBC (10^3^/mm^3^)	5.7 (5.1–6.9)	5.8 (4.9–7.80)	5.6 (5.0–6.8)
RBC (10^6^/mm^3^)	4.5 ± 0.4	4.6 ± 0.35	4.4 ± 0.32 ^T††^
HGB (g/dL)	13.8 ± 1.1	14.4 ± 0.1	13.2 ± 0.8 ^T††^
HCT (%)	39.7 (37.8–41.4)	40.9 (39.2–42.7)	38.8 (36.4–40.3) ^U††^
PLT (10^3^/mm^3^)	209.0 (179.7–241.2)	197.0 (168.0–231.0)	223.4 (891.0–243.0) ^U#^
MPV (µm^3^)	11.5 ± 1.0	11.3 ± 0.9	11.6 ± 1.1 ^T^*
PCT (%)	0.2 (0.2–0.3)	0.2 (0.2–0.3)	0.3 (0.2–0.3) ^U††^
Lym (10^3^/mm^3^)	1.9 (1.5–2.3)	1.7 (1.5–2.23)	1.9 (1.5–2.4)
Mono (10^3^/mm^3^)	0.5 (0.4–0.6)	0.6 (0.5–0.7)	0.5 (0.4–0.6) ^U#^
Neu (10^3^/mm^3^)	3.0 (2.4–3.9)	3.6 (2.5–3.8)	2.9 (2.4–4.0)
Eo (10^3^/mm^3^)	0.1 (0.09–0.2)	0.2 (0.1–0.2)	0.1 (0.09–0.21)
Baso (10^3^/mm^3^)	0.02 (0.02–0.03)	0.03 (0.02–0.04)	0.02 (0.02–0.03)
Total cholesterol (mmol/L)	5.4 (4.6–6.2)	4.8 (4.4–5.6)	5.8 (4.9–6.5) ^U††^
Triglycerides (mmol/L)	1.3 (0.9–1.8)	1.3 (0.9–1.7)	1.3 (0.9–4.2)
HDL cholesterol (mmol/L)	1.3 (1.1–1.6)	1.2 (1.0–1.4)	1.4 (1.1–1.7) ^U††^
LDL cholesterol (mmol/L)	3.4 (2.8–4.0)	3.0 (2.6–3.7)	3.7 (2.9–4.3) ^U††^
Glucose (mmol/L)	5.5 (5.0–5.6)	5.6 (5.2–6.3)	5.3 (4.9–5.9) ^U#^
Uric acid (mmol/L)	0.3 ± 0.07	0.3 ± 0.06	0.2 ± 1.3 ^T††^
Testosterone (ng/mL)	3.9 (2.9–4.5)	4.5 (4.4.–4.7)	2.9 (2.8–3.0) ^U††^
Dihydrotestosterone (ng/mL)	0.5 (0.3–0.6)	0.63 (0.61–0.66)	0.32 (0.31–0.33) ^U††^

Variables (not adjusted) presented as means ± SD or medians with interquartile ranges (from lower [25%] to upper [75%] quartile). Comparisons between men and women were performed with the use of the unpaired Student’s *t*-test (^T^) or Mann–Whitney *U* test (^U^). * *p* ≤ 0.05, ^#^ *p* < 0.01, ^††^ *p* < 0.0001. Abbreviations used: Baso, number of basophils; BMI, body mass index; Eo, number of eosinophils; HCT, hematocrit; HDL, high-density lipoproteins; HGB, concentration of hemoglobin; LDL, low-density lipoproteins; LYM, number of lymphocytes; Mono, number of monocytes; MPV, mean platelet volume; Neu, number of neutrophils; PCT, plateletcrit; PLT, platelet count; RBC, red blood cell count; WBC, white blood cell count; WHR, waist–hip ratio.

**Table 2:** Numbers and percentages of men and women whose GDS score was indicative of depression.

Answers Suggesting Depression	Number (Percentage) of Men with the Expected Answer, %	Number (Percentage) of Women with the Expected Answer, %
1 (No)	62 (89)	59 (78)
2 (Yes)	25 (35)	32 (41)
3 (Yes)	12 (17)	23 (30)
4 (Yes)	3 (4)	14 (19)
5 (No)	62 (87)	71 (93)
6 (Yes)	20 (28)	35 (46)
7 (No)	62 (89)	60 (79)
8 (Yes)	20 (7)	18 (24)
9 (Yes)	26 (37)	23 (30)
10 (Yes)	13 (18)	14 (19)
11 (No)	70 (99)	73 (95)
12 (Yes)	3 (4)	2 (4)
13 (No)	59 (83)	60 (79)
14 (Yes)	1 (1)	5 (6)
15 (Yes)	13 (20)	20 (26)

Variables presented as numbers per 71 men or 79 women (and percentages) of men or women who gave the answer suggesting depression (attributed one point, “1”) in response to particular questions of GDS scale. We indicated which answer is suggesting depression (attributed one point, “1”) in a quaestionnaire by entering “yes” or “no” next to the number of each individual question.

**Table 3:** Relationships between GDS scores and sex of the studied individuals.

Question No.	Question	Chi^2^ Test		Fisher’s Exact Test
		*X* ^2^	*p*	*p*
1	Are you satisfied with your entire life?	5.087	0.024	
2	Have you dropped many activities or have your interests diminished?	5.087	0.024	
3	Do you feel that your life is empty?	5.182	0.023	
4	Do you often get bored?	9.214	0.002	
5	Are you in good spirits most of the time?	1.700	0.192	
6	Are you afraid that something bad is going to happen to you?	7.024	0.008	
7	Do you feel happy most of the time?	4.113	0.043	
8	Do you often feel helpless?	9.262	0.002	
9	Do you prefer to stay at home, rather than going out and doing things?	1.354	0.245	
10	Do you feel that you have more problems with your memory than most?	0.846	0.358	
11	Do you think it is wonderful to be alive now?	1.453	0.228	
12	Do you feel worthless the way you are now?			0.723
13	Do you feel full of energy?	1.136	0.286	
14	Do you feel that your situation is hopeless?			0.121
15	Do you think that most people are better off than you are?	2.136	0.144	

Relationships between the GDS scores and sex were calculated with the bootstrap-boosted chi-squar test (10,000 iterations) with the size adjustment of 78 in each sex subgroup. Where the expected frequencies were too small (or at the boundary limits), the bootstrap-boosted Fisher’s exact test was used instead of the chi-squar test. Two-sided *p*-values (by the summation) are given for significance.

**Table 4:** Associations between plasma testosterone and dihydrotestosterone levels and GDS scores (total score and according to individual questions indicating potential depression).

GDS Questions	Both Sexes	Men	Women
1. Are you basically satisfied with your life?	0.055 ^TST, n.s.^	−0.002 ^TST, n.s.^	−0.159 ^TST,^ *
	0.065 ^DHT, n.s.^	−0.015 ^DHT, n.s.^	−0.002 ^DHT, n.s.^
2. Have your interests diminished?	−0.097 ^TST, n.s.^	−0.022 ^TST, n.s.^	−0.130 ^TST, n.s.^
	−0.126 ^DHT,^ *	−0.071 ^DHT, n.s.^	−0.192 ^DHT,^ *
3. Do you feel that your life is empty?	−0.097 ^TST, n.s.^	−0.085 ^TST, n.s.^	0.098 ^TST, n.s.^
	−0.114 ^DHT,^ *	−0.067 ^DHT, n.s.^	0.030 ^DHT, n.s.^
4. Do you often get bored?	−0.229 ^TST, #^	−0.144 ^TST, n.s.^	−0.061 ^TST, n.s.^
	−0.221 ^DHT, #^	−0.139 ^DHT, n.s.^	−0.040 ^DHT, n.s.^
5. Are you in good spirits most of the time?	−0.123 ^TST,^ *	−0.224 ^TST, #^	0.007 ^TST, n.s.^
	−0.121 ^DHT,^ *	−0.243 ^DHT, #^	0.037 ^DHT, n.s.^
6. Are you afraid that something bad is going to happen to you?	−0.086 ^TST, n.s.^	0.116 ^TST, n.s.^	0.062 ^TST, n.s.^
	−0.092 ^DHT, n.s.^	0.054 ^DHT, n.s.^	0.084 ^DHT, n.s.^
7. Do you feel happy most of the time?	0.038 ^TST, n.s.^	−0.099 ^TST, n.s.^	−0.111 ^TST, n.s.^
	0.042 ^DHT, n.s.^	−0.197 ^DHT, n.s.^	−0.092 ^DHT, n.s.^
8. Do you often feel helpless?	−0.238 ^TST, ##^	−0.036 ^TST, n.s.^	−0.192 ^TST,^ *
	−0.230 ^DHT, ##^	−0.027 ^DHT, n.s.^	−0.172 ^DHT, n.s.^
9. Do you prefer to stay at home?	0.079 ^TST, n.s.^	0.173 ^TST, #^	−0.061 ^TST, n.s.^
	−0.060 ^DHT, n.s.^	0.119 ^DHT, n.s.^	−0.082 ^DHT, n.s.^
10. Do you think you have more problems with your memory than most?	−0.019 ^TST, n.s.^	0.161 ^TST,^ *	−0.160 ^TST,^ *
	−0.025 ^DHT, n.s.^	0.075 ^DHT, n.s.^	−0.114 ^DHT, n.s.^
11. Do you think it is wonderful to be alive now?	0.081 ^TST, n.s.^	*nd* ^&^	−0.086 ^TST, n.s.^
	0.071 ^DHT, n.s.^		−0.114 ^DHT, n.s.^
12. Do you feel worthless the way you are now?	−0.027 ^TST, n.s.^	−0.031 ^TST, n.s.^	−0.096 ^TST, n.s.^
	−0.003 ^DHT, n.s.^	−0.018 ^DHT, n.s.^	−0.019 ^DHT, n.s.^
13. Do you feel full of energy?	0.054 ^TST, n.s.^	−0.022 ^TST, n.s.^	0.070 ^TST, n.s.^
	0.0308 ^DHT, n.s.^	−0.048 ^DHT, n.s.^	0.033 ^DHT, n.s.^
14. Do you feel that your situation is hopeless?	−0.149 ^TST, #^	−0.100 ^TST, n.s.^	−0.083 ^TST, n.s.^
	−0.146 ^DHT, #^	−0.092 ^DHT, n.s.^	−0.078 ^DHT, n.s.^
15. Do you think that most people are better off than you are?	−0.033 ^TST, n.s.^	−0.108 ^TST, n.s.^	0.126 ^TST, n.s.^
	−0.027 ^DHT, #^	−0.080 ^DHT, n.s.^	0.127 ^DHT, n.s.^
Total GDS score	−0.151 ^TST, #^	0.136 ^TST, n.s.^	−0.010 ^TST, n.s.^
	−0.165 ^DHT, #^	0.099 ^DHT, n.s.^	−0.026 ^DHT, n.s.^

Analysis of the correlation between the plasma testosterone (TST) and dihydrotestosterone (DHT) and GDS scores obtained in the GDS questionnaire (single questions and total score) among all patients, men and women. Results are shown as Spearman’s rank correlation coefficients. The coefficients of correlations with a statistical significance, i.e., *p* < 0.05 or *p* < 0.01, are indicated by ^#^ or ^##^, respectively. The statistical tendency (*p* = 0.05 or 0.05 < *p* < 0.1) is indicated with *. Statistically insignificant coefficients are marked ‘n.s.’. Among the men, ^&^ nd, not determined/not calculated because of none or single negative responses (100% or 98.63% of the “1” responses) in the subgroup.

**Table 5:** Point-biserial correlation coefficients for the answers suggesting depression to singular questions of the GDS questionnaire and the plasma levels of testosterone and dihydrotestosterone.

GDS Questions	Both Sexes
1. Are you basically satisfied with your life?	0.067 ^TST, n.s.^
	0.080 ^DHT, n.s.^
2. Have you dropped many activities or have your interests diminished?	−0.131 ^TST, n.s.^
	−0.131 ^DHT, n.s.^
3. Do you feel that your life is empty?	−0.123 ^TST,^ *
	−0.124 ^DHT,^ *
4. Do you often get bored?	−0.253 ^TST, ###^
	−0.238 ^DHT, ###^
5. Are you in good spirits most of the time?	−0.058 ^TST, n.s.^
	−0.072 ^DHT, n.s.^
6. Are you afraid that something bad is going to happen to you?	−0.102 ^TST, n.s.^
	−0.114 ^DHT, n.s.^
7. Do you feel happy most of the time?	0.081 ^TST, n.s.^
	0.082 ^DHT, n.s.^
8. Do you often feel helpless?	−0.218 ^TST, ##^
	−0.213 ^DHT, #^
9. Do you prefer to stay at home, rather than going out and doing things?	0.061 ^TST, n.s.^
	0.051 ^DHT, n.s.^
10. Do you feel that you have more problems with your memory than most?	−0.043 ^TST, n.s.^
	−0.038 ^DHT, n.s.^
11. Do you think it is wonderful to be alive now?	0.119 ^TST, ##^
	0.129 ^DHT, ##^
12. Do you feel worthless the way you are now?	−0.014 ^TST, n.s.^
	−0.008 ^DHT, n.s.^
13. Do you feel full of energy?	0.071 ^TST, n.s.^
	0.056 ^DHT, n.s.^
14. Do you feel that your situation is hopeless?	−0.145 ^TST, ##^
	−0.143 ^DHT, #^
15. Do you think that most people are better off than you are?	−0.030 ^TST, n.s.^
	−0.033 ^DHT, n.s.^

Analysis of the correlation between the plasma level of testosterone (TST) and dihydrotestosterone (DHT) and the sum of points obtained for single questions among all the respondents (*n* = 150), except for cases treated with antidepressant drugs (five cases). Results are shown as point-biserial correlation coefficients. The coefficients of correlations with a statistical significance, i.e., *p* < 0.05, *p* < 0.01 or *p* < 0.001 are indicated by ^#^, ^##^ or ^###^, respectively. The statistical tendency (*p* = 0.05 or 0.05 < *p* < 0.1) is indicated with *; statistically insignificant coefficients are marked ‘n.s.’.

**Table 6:** Quade’s indices and gamma correlation coefficients for the associations between plasma concentrations of testosterone and dihydrotestosterone and the total GDS scores.

Hormone		Quade’s Index	Gamma Correlation	*p*
testosterone	*not age-matched*	−0.115		0.034
	*PSM not adjusted*	−0.056		0.314
	*PSM adjusted for sex*	0.078		0.154
	*PSM adjusted for marital status*	−0.058		0.349
	*PSM adjusted for sex and marital status*	−0.052		0.366
	*PSM adjusted for ‘loneliness’ status*	0.083		0.197
	*PSM adjusted for sex and ‘loneliness’ status*	0.078		0.195
dihydrotestosterone	*not age-matched*	−0.122		0.023
	*PSM not adjusted*	−0.066		0.231
	*PSM adjusted for sex*	0.058		0.297
	*PSM adjusted for marital status*	−0.069		0.258
	*PSM adjusted for sex and marital status*	−0.063		0.275
	*PSM adjusted for ‘loneliness’ status*	0.060		0.361
	*PSM adjusted for sex and ‘loneliness’ status*	0.056		0.352
testosterone	*not age-matched*		−0.135	0.021
	*age-matched*		−0.095	0.118
dihydrotestosterone	*not age-matched*		−0.144	0.014
	*age-matched*		−0.105	0.085

Non-adjusted, sex-adjusted and sex and marital status-adjusted bootstrap-boosted Quade’s indices (10,000 iterations) were estimated for the original (not age-matched) groups (71 men and 79 women) and for the age-matched (PSM algorithm) whole population of men and women (*n* = 142) adjusted to the primary sample size (*n* = 150). Significance was estimated based on Quade’s index value and its standard error [39,40]. Gamma correlation coefficients were estimated for original (not age-matched) groups (71 men and 79 women) and PSM-age-matched groups (with the sample size adjusted to *n* = 150).

## Data Availability

The data presented in this study are available on request from the corresponding author. The data are not publicly available due to ethical reasons.

## Supplementary Materials

The following supporting information can be downloaded at: https://www.mdpi.com/article/10.3390/ijerph191912507/s1, Table S1: Relationships between GDS scores and sex of the studied individuals; Table S2: Differences in plasma levels of testosterone and dihydrotestosterone in the “No” or “Yes” responders to singular questions on the GDS questionnaire.
