# Supplementary material for: Relationships between Plasma Concentrations of Testosterone and Dihydrotestosterone and Geriatric Depression Scale Scores in Men and Women Aged 60–65 Years—A Multivariate Approach with the Use of Quade’s Test
Source: Int J Environ Res Public Health. 2022 Sep 30;19(19):12507. doi: 10.3390/ijerph191912507 (PMC9566053; doi:10.3390/ijerph191912507)
Supplement: Supplementary file 1 [file ijerph-19-12507-s001.zip › ijerph-1937005-supplementary.pdf]

Table S1. Relationships between GDS scores and sex of the studied individuals

no.	*question*	*adjusted for age* OR + 95%CI	*P*	*adjusted for Tst* OR + 95%CI	*P*	*adjusted for DHT* OR + 95%CI	*P*
1	Are you basically satisfied with your life?	2.744; 1.055-7.141	0.035	6.825; 1.412-32.98	0.019	8.156; 1.433-46.40	0.020
2	Have you dropped many activities and interests diminished?	0.868; 0.446-1.689	0.626	4.697; 0.732-30.10	0.104	20.71; 0.988-434.2	0.051
3	Do you feel that your life is empty?	0.450; 0.203-0.996	0.034	0.376; 0.091-1.545	0.169	0.321; 0.065-1.578	0.160
4	Do you often get bored?	0.171; 0.046-0.633	0.007	0.792; 0.058-10.82	0.857	0.627; 0.023-16.88	0.780
5	Are you in good spirits most of the time?	0.620; 0.188-2.046	0.360	0.323; 0.019-5.427	0.432	0.437; 0.019-9.693	0.600
6	Are you afraid that something bad is going to happen to you?	0.343; 0.167-0.706	0.003	0.133; 0.027-0.645	0.012	0.093; 0.013-0.659	0.017
7	Do you feel happy most of the time?	2.316; 0.882-6.077	0.077	4.271; 0.901-20.22	0.066	5.573; 1.004-30.91	0.049
8	Do you often feel helpless?	0.245; 0.084-0.709	0.003	0.323; 0.050-2.076	0.213	0.255; 0.034-1.870	0.171
9	Do you prefer to stay at home, rather than going out and doing things?	1.355; 0.682-2.693	0.461	1.547; 0.395-6.057	0.539	2.223; 0.385-12.81	0.374
10	Do you feel that you have more problems with memory than most?	1.046; 0.448-2.440	0.865	2.124; 0.299-15.09	0.484	2.836; 0.207-38.73	0.454
11	Do you think it is wonderful to be alive now?	*nd*[&]		*nd*[&]	0.997	43839; 0-0	0.997
12	Do you feel worthless the way you are now?	0.626; 0.123-3.168	0.349	2.390; 0.040-140.9	0.754	3.293; 0.008-1353.	0.744
13	Do you feel full of energy?	1.258; 0.536-2.951	0.440	0.644; 0.094-4.378	0.683	0.815; 0.096-6.865	0.875
14	Do you feel that your situation is hopeless?	0.198; 0.022-1.779	0.105	0.865; 0.016-45.90	0.844	2.213; 0.004-997.9	0.875
15	Do you think that most people are better off than you are?	0.605; 0.278-1.314	0.204	0.368; 0.092-1.472	0.164	0.303; 0.062-1.461	0.143

The relationships between the GDS scores and sex calculated with the use of bootstrap-boosted multivariate logistic regression with the adjustment either for age or for age and Tst/DHT concentrations (n=150). The outcomes of the logistic regression analysis are presented as the OR + 95%CI, P for each of the questions from the GDS questionnaire. In the analysis women constituted the reference class, men were the modelled class; in the analysis we used the V-fold cross validation. *nd*[&], not determined/not calculated because of none or single negative responses (100% or 98.63% of the “1” responses) in the subgroup of men.

Table S2. Differences in plasma levels of testosterone and dihydrotestosterone in the responders of "No" or "Yes" to singular questions of GDS questionnaire

GDS questions	Both sexes	Males	Females
1. Are you basically satisfied with your life? no	^{Tst}(Yes): 3.00; 2.91-4.40<br>^{Tst}(No): 4.31; 2.94-4.50<br>$P_{bootstrap\ ANCOVA–adjusted\ for\ age\ and\ sex}$ = 0.125<br>^{DHT}(Yes): 0.33; 0.32-0.62<br>^{DHT}(No):0.61; 0.32-0.63<br>$P_{bootstrap\ ANCOVA–adjusted\ for\ age\ and\ sex}$ = 0.105	^{Tst}(Yes): 4.48; 4.45-4.60<br>^{Tst} (No): 4.49; 4.38-4.71<br>$P_{bootstrap\ ANCOVA–adjusted\ for\ age\ and\ size}$ = 0.653<br>^{DHT}(Yes): 0.63l 0.63-0.65<br>^{DHT}(No): 0.63; 0.62-0.66<br>$P_{bootstrap\ ANCOVA–adjusted\ for\ age\ and\ size}$ = 0.772	^{Tst}(Yes): 2.94; 2.89-3.03<br>^{Tst} (No): 0.293; 2.86-3.00<br>$P_{bootstrap\ ANCOVA–adjusted\ for\ age\ and\ size}$ = 0.092<br>^{DHT}(Yes): 0.32; 0.32-0.33<br>^{DHT}(No): 0.32; 0.,32-0.33<br>$P_{bootstrap\ ANCOVA–adjusted\ for\ age\ and\ size}$ = 0.097
2. Have you dropped many activities and interests diminished? yes	^{Tst}(No): 4.32; 2.94-4.50<br>^{Tst} (Yes): 3.11; 2.90-4.46<br>$P_{bootstrap\ ANCOVA–adjusted\ for\ age\ and\ sex}$ = 0.039<br>^{DHT}(No): 0.61; 0.32-0.64<br>^{DHT}(Yes): 0.33; 0.32-0.63<br>$P_{bootstrap\ ANCOVA–adjusted\ for\ age\ and\ sex}$ = 0.020	^{Tst}(No): 4.49; 4.41-4.66<br>^{Tst} (Yes): 4.48; 4.38-4.71<br>$P_{bootstrap\ ANCOVA–adjusted\ for\ age\ and\ size}$ = 0.389<br>^{DHT}(No): 0.63; 0.62-0.66<br>^{DHT}(Yes): 0.63; 0.62-0.66<br>$P_{bootstrap\ ANCOVA–adjusted\ for\ age\ and\ size}$ = 0.204	^{Tst}(No): 2.94; 2.88-3.03<br>^{Tst} (Yes): 2.94; 2.83-2.99<br>$P_{bootstrap\ ANCOVA–adjusted\ for\ age\ and\ size}$ = 0.060<br>^{DHT}(No): 0.32; 0.32-0.34<br>^{DHT}(Yes): 0.32; 0.31-0.33<br>$P_{bootstrap\ ANCOVA–adjusted\ for\ age\ and\ size}$ = 0.047
3. Do you feel that your life is empty? yes	^{Tst}(No): 4.32; 2.94-4.51<br>^{Tst} (Yes): 3.08; 2.88-4.36<br>$P_{bootstrap\ ANCOVA–adjusted\ for\ age\ and\ sex}$ = 0.814<br>^{DHT}(No): 0.61; 0.32-0.64<br>^{DHT}(Yes): 0.34; 0.32-0.61<br>$P_{bootstrap\ ANCOVA–adjusted\ for\ age\ and\ sex}$ = 0.671	^{Tst}(No): 4.49; 4.42-4.71<br>^{Tst} (Yes): 4.47; 4.36-4.58<br>$P_{bootstrap\ ANCOVA–adjusted\ for\ age\ and\ size}$ = 0.369<br>^{DHT}(0No): 0.63; 0.62-0.66<br>^{DHT}(Yes): 0.63; 0.62-0.65<br>$P_{bootstrap\ ANCOVA–adjusted\ for\ age\ and\ size}$ = 0.496	^{Tst}(No): 2.94; 2.86-2.99<br>^{Tst} (Yes): 2.94; 2.84-3.08<br>$P_{bootstrap\ ANCOVA–adjusted\ for\ age\ and\ size}$ = 0.532<br>^{DHT}(No): 0.32; 0.32-0.33<br>^{DHT}(Yes): 0.32; 0.32-0.34<br>$P_{bootstrap\ ANCOVA–adjusted\ for\ age\ and\ size}$ = 0.491
4. Do you often get bored? yes	^{Tst}(No): 4.32; 294-4.52<br>^{Tst} (Yes): 2.94; 2.83-3.16<br>$P_{bootstrap\ ANCOVA–adjusted\ for\ age\ and\ sex}$ = 0.136<br>^{DHT}(No): 0.61; 0.32-0.64<br>^{DHT}(Yes): 0.32; 0.31-0.34	^{Tst}(No): 4.50; 4.41-4.71<br>^{Tst} (Yes): 4.40; 4.38-4.44<br>$P_{bootstrap\ ANCOVA–adjusted\ for\ age\ and\ size}$ = 0.327<br>^{DHT}(No): 0.63; 0.62-0.66<br>^{DHT}(Yes): 0.62; 0.62-0.63	^{Tst}(No): 2.94; 2.86-3.01<br>^{Tst} (Yes): 2.90; 2.82-3.01<br>$P_{bootstrap\ ANCOVA–adjusted\ for\ age\ and\ size}$ = 0.268<br>^{DHT}(No): 0.32; 0.32-0.33<br>^{DHT}(Yes): 0.32; 0.31-0.33

	$P_{\text{bootstrap ANCOVA–adjusted for age and sex}}$ = 0.293	$P_{\text{bootstrap ANCOVA–adjusted for age and size}}$ = 0.382	$P_{\text{bootstrap ANCOVA–adjusted for age and size}}$ = 0.459
5. Are you in good spirits most of the time? no	^{Tst}(Yes): 4.47; 3.05-4.72<br>^{Tst} (No): 3.34; 2.93-4.48<br>$P_{\text{bootstrap ANCOVA–adjusted for age and sex}}$ = 0.688<br>^{DHT}(Yes): 0.63; 0.34-0.67<br>^{DHT}(No): 0.63; 0.32-0.63<br>$P_{\text{bootstrap ANCOVA–adjusted for age and sex}}$ = 0.855	^{Tst}(Yes): 4.64; 4.48-4.91<br>^{Tst} (No): 4.48; 4.36-4.64<br>$P_{\text{bootstrap ANCOVA–adjusted for age and size}}$ = 0.135<br>^{DHT}(Yes): 0.66; 0.63-0.69<br>^{DHT}(No): 0.63; 0.62-0.65<br>$P_{\text{bootstrap ANCOVA–adjusted for age and size}}$ = 0.100	^{Tst}(Yes): 2.94; 2.87-3.05<br>^{Tst} (No): 2.94; 2.86-3.01<br>$P_{\text{bootstrap ANCOVA–adjusted for age and size}}$ = 0.118<br>^{DHT}(Yes): 0.32; 0.32-0.34<br>^{DHT}(No): 0.32; 0.32-0.33<br>$P_{\text{bootstrap ANCOVA–adjusted for age and size}}$ = 0.216
6. Are you afraid that something bad is going to happen to you? yes	^{Tst}(No): 4.33; 2.94-4.48<br>^{Tst} (Yes): 3.05; 2.89-4.47<br>$P_{\text{bootstrap ANCOVA–adjusted for age and sex}}$ = 0.138<br>^{DHT}(No): 0.61; 0.32-0.63<br>^{DHT}(Yes): 0.33; 0.32-0.63<br>$P_{\text{bootstrap ANCOVA–adjusted for age and sex}}$ = 0.111	^{Tst}(No): 4.48; 4.37-4.62<br>^{Tst} (Yes): 4.54; 4.45-4.80<br>$P_{\text{bootstrap ANCOVA–adjusted for age and size}}$ = 0.663<br>^{DHT}(No): 0.63; 0.62-0.65<br>^{DHT}(Yes): 0.64; 0.62-0.67<br>$P_{\text{bootstrap ANCOVA–adjusted for age and size}}$ = 0.858	^{Tst}(No): 2.94; 2.87-2.97<br>^{Tst} (Yes): 2.92; 2.86-3.05<br>$P_{\text{bootstrap ANCOVA–adjusted for age and size}}$ = 0.160<br>^{DHT}(No): 0.32; 0.32-0.33<br>^{DHT}(Yes): 0.32; 0.32-0.33<br>$P_{\text{bootstrap ANCOVA–adjusted for age and size}}$ = 0.080
7. Do you feel happy most of the time? no	^{Tst}(Yes): 3.04; 2.91-4.47<br>^{Tst} (No): 4.31; 2.94-4.49<br>$P_{\text{bootstrap ANCOVA–adjusted for age and sex}}$ = 0.321<br>^{DHT}(Yes): 0.33; 0.32-0.63<br>^{DHT}(No): 0.61; 0.32-0.63<br>$P_{\text{bootstrap ANCOVA–adjusted for age and sex}}$ = 0.198	^{Tst}(Yes): 4.56; 4.48-4.67<br>^{Tst} (No): 4.48; 4.38-4.69<br>$P_{\text{bootstrap ANCOVA–adjusted for age and size}}$ = 0.904<br>^{DHT}(Yes): 0.64; 0.63-0.66<br>^{DHT}(No): 0.63; 0.62-0.66<br>$P_{\text{bootstrap ANCOVA–adjusted for age and size}}$ = 0.864	^{Tst}(Yes): 2.94; 2.89-3.05<br>^{Tst} (No): 2.94; 2.86-2.99<br>$P_{\text{bootstrap ANCOVA–adjusted for age and size}}$ = 0.316<br>^{DHT}(Yes): 0.32; 0.32-0.34<br>^{DHT}(No): 0.32; 0.32-0.33<br>$P_{\text{bootstrap ANCOVA–adjusted for age and size}}$ = 0.223
8. Do you often feel helpless? yes	^{Tst}(No): 4.32; 2.95-4.50<br>^{Tst} (Yes): 2.94; 2.84-3.03<br>$P_{\text{bootstrap ANCOVA–adjusted for age and sex}}$ = 0.576<br>^{DHT}(No): 0.61; 0.32-0.63<br>^{DHT}(Yes): 0.32; 0.31-0.33	^{Tst}(No): 4.48; 4.40-4.70<br>^{Tst} (Yes): 4.52; 4.40-4.56<br>$P_{\text{bootstrap ANCOVA–adjusted for age and size}}$ = 0.544<br>^{DHT}(No): 0.63; 0.62-0.66<br>^{DHT}(Yes): 0.64; 0.62-0.64	^{Tst}(No): 2.94; 2.87-3.02<br>^{Tst} (Yes): 2.92;2.82-2.94<br>$P_{\text{bootstrap ANCOVA–adjusted for age and size}}$ = 0.722<br>^{DHT}(No): 0.32; 0.32-0.33<br>^{DHT}(Yes): 0.32; 0.31-0.32

	$P_{\text{bootstrap ANCOVA–adjusted for age and sex}}$ = 0.823	$P_{\text{bootstrap ANCOVA–adjusted for age and size}}$ = 0.615	$P_{\text{bootstrap ANCOVA–adjusted for age and size}}$ = 0.896
9. Do you prefer to stay at home, rather than going out and doing things? yes	^{Tst}(No): 3.24; 2.94-4.47<br>^{Tst} (Yes): 4.29; 2.93-4.56<br>$P_{\text{bootstrap ANCOVA–adjusted for age and sex}}$ = 0.789<br>^{DHT}(No): 0.35; 0.32-0.63<br>^{DHT}(Yes): 0.57; 0.32-0.64<br>$P_{\text{bootstrap ANCOVA–adjusted for age and sex}}$ = 0.520	^{Tst}(No): 4.48; 4.36-4.58<br>^{Tst} (Yes): 4.56; 4.42-4.77<br>$P_{\text{bootstrap ANCOVA–adjusted for age and size}}$ = 0.063<br>^{DHT}(No): 0.63; 0.62-0.65<br>^{DHT}(Yes): 0.64; 0.62-0.67<br>$P_{\text{bootstrap ANCOVA–adjusted for age and size}}$ = 0.158	^{Tst}(No): 2.94; 2.86-3.03<br>^{Tst} (Yes): 2.93; 2.88-2.96<br>$P_{\text{bootstrap ANCOVA–adjusted for age and size}}$ = 0.183<br>^{DHT}(No): 0.32; 0.32-0.33<br>^{DHT}(Yes): 0.32; 0.32-0.32<br>$P_{\text{bootstrap ANCOVA–adjusted for age and size}}$ = 0.157
10. Do you feel that you have more problems with memory than most? yes	^{Tst}(No): 3.97; 2.94-4.48<br>^{Tst} (Yes): 2.96; 2.89-4.49<br>$P_{\text{bootstrap ANCOVA–adjusted for age and sex}}$ = 0.387<br>^{DHT}(No): 0.50; 0.32-0.63<br>^{DHT}(Yes): 0.33; 0.32-0.63<br>$P_{\text{bootstrap ANCOVA–adjusted for age and sex}}$ = 0.387	^{Tst}(No): 4.48; 4.36-4.68<br>^{Tst} (Yes): 4.52; 4.46-4.72<br>$P_{\text{bootstrap ANCOVA–adjusted for age and size}}$ = 0.846<br>^{DHT}(No): 0.63; 0.62-0.66<br>^{DHT}(Yes): 0.63; 0.63-0.65<br>$P_{\text{bootstrap ANCOVA–adjusted for age and size}}$ = 0.469	^{Tst}(No): 2.94; 2.86-3.02<br>^{Tst} (Yes): 2.91; 2.85-2.94<br>$P_{\text{bootstrap ANCOVA–adjusted for age and size}}$ = 0.295<br>^{DHT}(No): 0.32; 0.32-0.33<br>^{DHT}(Yes): 0.32; 0.31-0.32<br>$P_{\text{bootstrap ANCOVA–adjusted for age and size}}$ = 0.533
11. Do you think it is wonderful to be alive now? no	^{Tst}(Yes): 2.94; 2.94-3.00<br>^{Tst} (No): 4.29; 2.93-4.48<br>$P_{\text{bootstrap ANCOVA–adjusted for age and sex}}$ = 0.940<br>^{DHT}(Yes): 0.32; 0.32-0.33<br>^{DHT}(No): 0.57; 0.32-0.63<br>$P_{\text{bootstrap ANCOVA–adjusted for age and sex}}$ = 0.835	*nd*[&]<br>^{Tst}(Yes): -<br>^{Tst} (No): 4.48; 4.40-4.70<br>^{DHT}(Yes): -<br>^{DHT}(No): 0.63; 0.62-0.66	^{Tst}(Yes): 2.94; 2.94-3.00<br>^{Tst} (No): 2.94; 2.86-3.01<br>$P_{\text{bootstrap ANCOVA–adjusted for age and size}}$ = 0.904<br>^{DHT}(Yes): 0.32; 0.32-0.33<br>^{DHT}(No): 0.32; 0.32-0.33<br>$P_{\text{bootstrap ANCOVA–adjusted for age and size}}$ = 0.804
12. Do you feel worthless the way you are now? yes	^{Tst}(No): 4.28; 2.04-4.49<br>^{Tst} (Yes): 2.94; 2.87-4.42<br>$P_{\text{bootstrap ANCOVA–adjusted for age and sex}}$ = 0.499<br>^{DHT}(No): 0.54; 0.32-63<br>^{DHT}(1Yes): 0.33; 0.32-0.62	^{Tst}(No): 4.49; 4.38-4.71<br>^{Tst} (Yes): 4.47; 4.44-4.53<br>$P_{\text{bootstrap ANCOVA–adjusted for age and size}}$ = 0.561<br>^{DHT}(No): 0.63; 0.62-0.66<br>^{DHT}(Yes): 0.63; 0.63-0.64	^{Tst}(No): 2.94; 2.86-3.02<br>^{Tst} (Yes): 2.89; 2.82-2.93<br>$P_{\text{bootstrap ANCOVA–adjusted for age and size}}$ = 0.637<br>^{DHT}(No): 0.32; 0.32-0.33<br>^{DHT}(Yes): 0.32; 0.31-0.32

	$P_{\text{bootstrap ANCOVA–adjusted for age and sex}}$ = 0.546	$P_{\text{bootstrap ANCOVA–adjusted for age and size}}$ = 0.631	$P_{\text{bootstrap ANCOVA–adjusted for age and size}}$ = 0.656
13. Do you feel full of energy? no	^{Tst}(Yes): 3.24; 2.89-4.47<br>^{Tst} (No): 4.30; 2.94-4.48<br>$P_{\text{bootstrap ANCOVA–adjusted for age and sex}}$ = 0.405<br>^{DHT}(Yes): 0.35; 0.32-0.63<br>^{DHT}(No): 0.57; 0.32-0.63<br>$P_{\text{bootstrap ANCOVA–adjusted for age and sex}}$ = 0.616	^{Tst}(Yes): 4.51; 4.42-4.65<br>^{Tst} (No): 4.48; 4.37-4.70<br>$P_{\text{bootstrap ANCOVA–adjusted for age and size}}$ = 0.758<br>^{DHT}(Yes): 0.64; 0.62-0.66<br>^{DHT}(No): 0.63; 0.62-0.66<br>$P_{\text{bootstrap ANCOVA–adjusted for age and size}}$ = 0.894	^{Tst}(Yes): 2.90; 2.83-3.05<br>^{Tst} (No): 2.94; 2.86-3.01<br>$P_{\text{bootstrap ANCOVA–adjusted for age and size}}$ = 0.437<br>^{DHT}(Yes): 0.32; 0.31-0.34<br>^{DHT}(No): 0.32; 0.32-0.33<br>$P_{\text{bootstrap ANCOVA–adjusted for age and size}}$ = 0.603
14. Do you feel that your situation is hopeless? yes	^{Tst}(No): 4.30; 2.94-4.49<br>^{Tst} (Yes): 2.94; 2.86-3.00<br>$P_{\text{bootstrap ANCOVA–adjusted for age and sex}}$ = 0.359<br>^{DHT}(No): 0.60; 0.32-0.63<br>^{DHT}(Yes): 0.32; 0.32-0.33<br>$P_{\text{bootstrap ANCOVA–adjusted for age and sex}}$ = 0.392	^{Tst}(No): 4.49; 4.40-4.70<br>^{Tst} (Yes): 4.40; 4.40-4.40<br>$P_{\text{bootstrap ANCOVA–adjusted for age and size}}$ = 0.552<br>^{DHT}(No): 0.63; 0.62-0.66<br>^{DHT}(Yes): 0.62; 0.62-0.62<br>$P_{\text{bootstrap ANCOVA–adjusted for age and size}}$ = 0.587	^{Tst}(No): 2.94; 2.86-3.01<br>^{Tst} (Yes): 2.92; 2.84-2.94<br>$P_{\text{bootstrap ANCOVA–adjusted for age and size}}$ = 0.491<br>^{DHT}(No): 0.32; 0.32-0.33<br>^{DHT}(Yes): 0.32; 0.32-0.32<br>$P_{\text{bootstrap ANCOVA–adjusted for age and size}}$ = 0.519
15. Do you think that most people are better off than you are? yes	^{Tst}(No): 4.32; 2.94-4.49<br>^{Tst} (Yes): 3.05; 2.92-4.40<br>$P_{\text{bootstrap ANCOVA–adjusted for age and sex}}$ = 0.392<br>^{DHT}(No): 0.61; 0.32-0.63<br>^{DHT}(Yes): 0.34; 0.32-0.62<br>$P_{\text{bootstrap ANCOVA–adjusted for age and sex}}$ = 0.306	^{Tst}(No): 4.48; 4.42-4.72<br>^{Tst} (Yes): 4.50; 4.36-4.58<br>$P_{\text{bootstrap ANCOVA–adjusted for age and size}}$ = 0.248<br>^{DHT}(No): 0.63; 0.62-0.66<br>^{DHT}(Yes): 0.64; 0.61-0.65<br>$P_{\text{bootstrap ANCOVA–adjusted for age and size}}$ = 0.374	^{Tst}(No): 2.94; 2.86-3.00<br>^{Tst} (Yes): 2.94; 2.87-3.02<br>$P_{\text{bootstrap ANCOVA–adjusted for age and size}}$ = 0.146<br>^{DHT}(No): 0.32; 0.32-0.33<br>^{DHT}(Yes): 0.32; 0.32-0.33<br>$P_{\text{bootstrap ANCOVA–adjusted for age and size}}$ = 0.148

Data presented as medians and interquartile ranges (Me; IQR). Differences in plasma levels of testosterone (TST) and dihydrotestosterone (DHT) in the responders of “No” or “Yes” to singular questions of GDS questionnaire in all patients and separate subgroups of males and females estimated with the bootstrap-boosted ANCOVA adjusted for age /and sex. In the separate sexual subgroups the ANCOVA adjusted to age and the overall sample size of a whole group studied (*N*=150). [&]nd, not determined/not calculated because of none or single negative responses (100% or 98.63% of the “1” responses) in the subgroup of men. In the first column we marked which response (“Yes” or “No”) has been attributed to the rank 1.
